# Profile of central corneal thickness and corneal endothelial cell morpho-density of in healthy Congolese eyes

**DOI:** 10.1186/s12886-021-01947-x

**Published:** 2021-04-22

**Authors:** Joseph-Theodore K. Kelekele, David L. Kayembe, Jean-Claude Mwanza

**Affiliations:** 1 Department of Ophthalmology, University Hospital of Kinshasa, Kinshasa, Democratic Republic of Congo; 2 Department of Ophthalmology, University of North Carolina at Chapel Hill, Chapel Hill, North Carolina USA

**Keywords:** Central corneal thickness, Corneal endothelial cell density, Healthy eyes, Specular microscopy

## Abstract

**Purpose:**

To determine the pachymetric and corneal endothelial cell morphometric features and their relationship to ocular and systemic factors in healthy Congolese subjects.

**Methods:**

Non-contact specular microscopy was used in 278 healthy eyes (278 subjects) to measure central corneal thickness (CCT), corneal endothelial cell density (CECD) along with cell size, coefficient of variation (CV) in cell size, and hexagonality (HEX). The lower and upper reference limits and average values for each parameter were determined. Correlation and association of average values with anthropo-demographic and clinical variables were assessed.

**Results:**

The mean age was 38.9 ± 17.2 years (10.9–80.7 years). Average values were 504.2 ± 30.7 μm (CCT), 2907.1 ± 290.9 cells/mm^2^ (CECD), 348.5 ± 38.4 μm^2^ (cell size), 32.9 ± 3.6% (CV), and 51.8 ± 7.2% (HEX). CCT was 504.9 ± 33.6 μm in men and 503.6 ± 28.3 μm in women (*p* = .73); values for CECD were 2917.1 ± 253.5 cells/mm^2^ and 2899.2 ± 317.8 cells/mm^2^ (*p* = 0.61), respectively. Lower and upper reference limits were 449.6 μm and 566.0 μm for CCT, and 2165.3 cells/mm^2^ and 3414.4 cells/mm^2^ for CECD, respectively. CCT correlated with body mass index (BMI), (*r* = − 0.12, *P* = 0.04). CECD decreased with age (*r* = − 0.49, *P* < 0.001), BMI (*r* = − 0.20, *P* = 0.001), intraocular pressure (*r* = − 0.13, *P* = 0.029) and ocular perfusion pressure (*r* = − 0.28, *P* = 0.028). CECD decayed by 8.3 cells/mm^2^ or 0.30% per year of age and CCT decreased by 0.72 μm per kg/m^2^.

**Conclusions:**

Mean central cornea was thinner, CECD higher, and references limits lower than reported in other African populations. The CCT and CECD normative values reported herein will be useful for both clinical and research purposes in this population.

**Supplementary Information:**

The online version contains supplementary material available at 10.1186/s12886-021-01947-x.

## Introduction

Assessment of anatomical organs for disease detection involves careful examination of their morphological and oftentimes their quantitative features. Since organ abnormalities are always defined as a departure from normal state, it is important to determine the characteristics of normalcy of each organ both morphologically and quantitatively. In the case of the cornea, central corneal thickness (CCT) as well as the morphology and density of corneal endothelial cells (CEC) are important parameters that have long been used for risk stratification, disease monitoring and assessment of treatment efficacy in several situations. These include, but are not limited to glaucoma, assessment of corneal ectasia, detection of corneal edema secondary to contact lens wear, and planning of keratorefractive surgery and corneal graft. Both CCT and corneal endothelial cell density (CECD) have been studied in different healthy populations and have been shown to be affected by factors such as genetics [1–3], race/ethnicity [4–8], and age [9–11]. The variability across races/ethnicities implies that normative values from one population may not be applicable to others. It also means it is recommended for each subpopulation to determine its own reference values for meaningful interpretation of data from diseased eyes.

Sub-Saharan Africa (SSA) is mostly racially homogenous, but ethnically very diverse. As a corollary, there may be differences in CCT and CECD across regions. However, data on CCT and CECD in normal eyes within SSA are only available for a few countries, precisely Nigeria [12, 13], South Africa [4, 14], Cameroon [15], Ghana [10, 16], Ethiopia [17], and Sudan [18]. A review of these reports suggests wide variations in these parameters within SSA sub-regions [19], further substantiating the establishment of normative values for each population or subpopulation. This view is also supported by consistent observation of thinner corneas in Blacks than other racial groups [4, 14, 20–22]. The purpose of this study was to determine the characteristics of CCT and corneal endothelial cell morpho-density in healthy Congolese eyes. These characteristics will be useful for reliable interpretation of data in this population.

## Subjects and methods

### Subjects

Participants of this study were 278 volunteers recruited among outpatients attending the Department of Ophthalmology of the University Hospital of Kinshasa, their accompanying family members and hospital staff. The study received approval from the Institutional Review Board of the School of Medicine, University of Kinshasa. Written consent was obtained from all adult participants and from parents of participant under 18 years of age. The study was conducted in compliance with the tenets of the Declaration of Helsinki. All participants were submitted to a standardized interview to collect information about any past or current ocular disease, trauma or surgery as well as systemic comorbidities that may affect CCT and/or CECD. Inclusion criteria for this study were being 10 years or older, having clear cornea and intraocular pressure (IOP) within normal range (10–21 mmHg). Those with a history or signs of previous intraocular (i.e. for cataract extraction, glaucoma) or corneal surgery, corneal inflammation, intraocular inflammation (i.e. uveitis), diabetes, chronic obstructive pulmonary disease, multiple transfusions or other underlying diseases or on treatment that may structurally affect the cornea were excluded from participation. There were no contact lens wearers.

### Clinical evaluation and central corneal thickness and corneal endothelium morphology and density assessment

Anthropometric and general clinical information was collected, including height (cm), weight (kg), and BMI calculated as $\frac{weight}{height^2}$, systolic blood pressure (SBP, mmHg), and diastolic blood pressure (DBP, mmHg). Participants were submitted to a routine ophthalmologic examination. This included best corrected visual acuity measurement, automated refraction (Topcon 9200, Tokyo, Japan) (expressed in diopters of spherical equivalent refraction (SER): $sphere+\frac{cylinder}{2}$), slit-lamp examination of the anterior segment, intraocular pressure (IOP) measurement by Goldmann applanation tonometry, and dilated direct ophthalmoscopy. The anterior segment was carefully examined for evidence of any sign suggestive of a congenital abnormality, structural abnormality such as obvious or occult keratoconus, signs of past or ongoing intraocular inflammation. Those found to have such anomalies were excluded from the study. Cup-to-disc ratio (CDR) was estimated during ophthalmoscopy.

The cornea was further evaluated with a digital Topcon SP-3000P noncontact specular microscope (Topcon Corporation, Tokyo, Japan) sequentially in each eye. Corneal parameters of interest were CCT (μm), CECD (cell/mm^2^), cell size (μm^2^), coefficient of variation (CV) in cell size, and hexagonality (%). CECD is the cell count in a 1 mm^2^ area. Hexagonality refers to the percentage of hexagon-like cells and is indicative of the degree of pleomorphism (cell shape variation). CV indicates the degree of polymegathism (cell size variability) and is derived from the formula: $\frac{standard\ deviation\ of\ mean\ size}{mean\ cell\ size}$. Values of these parameters were computed automatically by the device. All corneal measurements were obtained by the same examiner (JTK). For each eye, 3 measurements were obtained consecutively during the same session, separated one another by approximately 10 s during which the subject was instructed to pull his head back from device.

### Statistical analysis

Assuming that there won’t be a statistically significant difference between the average CCT of our Congolese sample (527.9 μm from an unpublished pilot study) and that from a population-based study in Ghana (533.9 ± 34.0 [10], at a 80% power, the formula $\mathrm{N}=\frac{\upsigma^2{\left({\mathrm{z}}_{1-\upbeta}+{\mathrm{z}}_{1-\upalpha /2}\right)}^2}{{\left({\upmu}_{\mathrm{o}}-{\upmu}_1\right)}^2}$ (where N = sample size of study population, σ = variance of study population, μ_0_ = population mean, μ_1_ = mean of study population, α = probability of type I error (usually 0.05), β = probability of type II error (usually 0.2), and z = critical Z value for a given α or β), suggested that we would need to study at least 252 subjects. Because of potential exclusion of subjects during data curation, a 10% attrition was added, bringing the total to 277 subjects.

Given the lack of difference between right and left eye in CCT (504.2 ± 30.7 vs. 505.2 ± 29.7, *p* = 0.32) and CECD (2907.1 ± 290.9 vs. 2909.6 ± 287.2, *p* = .76), only data of the right eye per participant was randomly selected was used for statistical analyses (SPSS version 20.0; SPSS, Chicago, IL, USA). The randomization was simply based on a single coin toss. One side of the coin was labelled right eye while the other was labelled left eye. The eye corresponding to the side facing up after the coin landed was retained as study eye for all participants. Because a small pilot analysis using data of the first 25 eyes showed a good repeatability (intraclass correlation coefficient (ICC) = 0.912 and a coefficient of variation (CV) = 1.4%), the average of the three measurements was used for all parameters. We used the Shapiro-Wilk (SW) test to assess the normality of the distribution. Failing the normality test (*p* < .05) indicates with 95% confidence that the data is not normally distributed, whereas passing it (*p* > .05) only suggests that there is no significant departure from normal distribution. Skewness was used as a measure of distribution’s symmetry. A skewness value between − 0.5 and + 0.5 was suggestive of a fairly symmetrical distribution, a value between − 1 and − 0.5 was indicative of a moderately skewed distribution whereas a value less than − 1 or greater than + 1 meant a highly skewed distribution. The 2.5th and 97.5th percentiles of CCT and CEC parameters were used to determine the lower and upper limits of normality, respectively. Values below the 2.5th percentiles were considered lower than normal; those between 2.5th and 97.5th percentiles were within normal range, and those beyond the 97.5th percentile were regarded as greater than normal. Differences in means between sexes and age groups were calculated with the unpaired Student t-test or analysis of variance as appropriate. Correlations of CCT and corneal epithelial cell parameters with age, IOP, CDR, SER, BMI, and mean ocular perfusion pressure (OPP, mmHg) were assessed using Pearson correlation. Mean OPP was calculated as: $\frac{2}{3} MAP- IOP$, where MAP or mean arterial pressure equals to $DBP+\frac{1}{3}\left( SBP- DBP\right)$. Both univariable and multivariable regression analyses were performed to assess the association of CCT and CECD with sex, age, height, weight, BMI, SER, IOP, CDR, SBP, DBP, and OPP, diabetes (yes/no), and smoking (yes/no). Statistical significance level was set at *p* < .05.

## Results

### Demographic and characteristics of study participants

A total of 280 subjects were enrolled, of whom 2 were subsequently excluded because of poor specular microscopic image quality. Demographic, general ocular and non-ocular features of the remaining 278 participants are shown in Table 1. The mean age was 38.9 ± 17.2 (minimum: 10.9; maximum: 80.7) years. There were significantly more females (56.1%) than males (*p* < .001). Men were significantly taller and had a lower BMI than females (all *p* < .001), but both were comparable in age, SER, IOP, CDR, SBP, DBP, OPP, and weight (all *p* > .05).

**Table 1:** Demographic and clinical characteristics of the study population

Parameter	Overall	Males vs. Females	*p* value
Number of subjects	278	122 vs. 156	0.004
Age, years	38.9 ± 17.2	39.6 ± 17.5 vs. 38.5 ± 16.9	0.61
Age by age band			
10–19 (*n* = 51)	16.8 ± 2.7	17.2 ± 2.8 vs. 16.6 ± 2.7	0.52
20–29 (*n* = 49)	24.5 ± 2.8	24.6 ± 2.9 vs. 24.5 ± 2.7	0.84
30–39 (*n* = 50)	35.0 ± 3.0	35.2 ± 2.8 vs. 38.9 ± 3.3	0.67
40–49 (*n* = 46)	45.6 ± 2.8	44.9 ± 2.7 vs. 46.3 ± 2.8	0.08
50–59 (*n* = 43)	53.7 ± 2.5	53.0 ± 2.1 vs. 54.0 ± 2.6	0.21
≥ 60 (*n* = 39)	67.3 ± 5.6	68.8 ± 6.4 vs. 65.8 ± 4.5	0.10
IOP, mmHg	14.3 ± 2.8	14.1 ± 2.7 vs. 14.5 ± 2.9	0.24
CDR	0.2 ± 0.1	0.2 ± 0.1 vs. 0.45 ± 0.15	0.09
OPP, mmHg	48.6 ± 8.9	48.3 ± 8.2 vs. 48.9 ± 9.4	0.60
SER, diopters	0.0 ± 1.3	0.0 ± 1.3 vs. 0.0 ± 1.4	0.91
Weight, kg	69.5 ± 15.4	69.1 ± 13.7 vs. 69.7 ± 16.6	0.75
Height, cm	167.9 ± 8.5	172.2 ± 7.7 vs. 164.6 ± 7.5	< 0.001
BMI	24.6 ± 5.2	23.3 ± 4.2 vs. 25.7 ± 5.6	< 0.001
SBP, mmHg	123.4 ± 18.4	123.8 ± 16.1 vs. 123.1 ± 20.1	0.75
DBP, mmHg	78.5 ± 12.9	77.4 ± 12.2 vs. 79.3 ± 13.5	0.24

*IOP* Intraocular pressure; *CDR* Cup-to-disc ratio; *OPP* Ocular perfusion pressure; *SER* Spherical equivalent refraction; *BMI* Body mass index; *SBP* Systolic blood pressure; *DBP* Diastolic blood pressure

### Central corneal thickness and corneal endothelium cell profiles

**Fig. 1:**
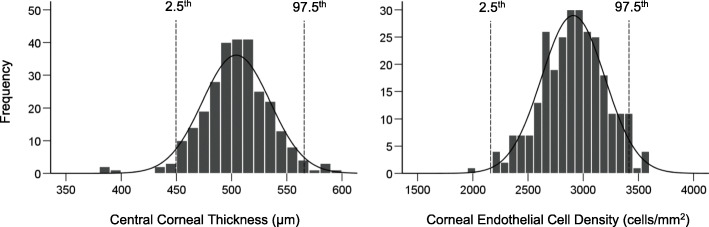
Frequency distribution of central corneal thickness (left) and corneal endothelial cell density (right) with lower (2.5th and upper 97.5th) reference limits lines

Table 2 presents the mean for CCT and corneal endothelial cell (CEC) parameters quantification in the entire study population and in the six age bands. Average CCT was 504.2 ± 30.7 μm (minimum: 382.7 μm, maximum: 590.7 μm); that of the CECD was 2907.1 ± 290.9 cells/mm^2^ (minimum: 1934.3 cells/mm^2^; maximum: 3621.0 cells/mm^2^). Using the definition of reference intervals corresponding to interval of values containing the central 95% of a healthy population, the 2.5th and 97.5th percentiles were 449.6 μm and 566.0 μm for CCT, and 2165.3 cells/mm^2^ and 3414.4 cells/mm^2^ for CECD, respectively (Fig. 1). Such values for cell size, CV and hexagonality are provided in Table 2. Figure 1 illustrates the frequency distribution of CCT and CECD in the study population, with reference lines for the 2.5th and 97.5th percentiles. Based on Shapiro-Wilk test, CCT, CECD, hexagonality, and cell size were not normally distributed whereas cell size showed normal distribution.

**Table 2:** Central corneal thickness and corneal endothelial cell characteristics in study participants stratified by age group

Age groups, percentiles, Shapiro-Wilk (SW), skewness	CCT	CECD	Cell size	CV	HEX
10–19 years	501.8 ± 27.3	3104.3 ± 246.9	324.8 ± 27.1	30.6 ± 3.7	55.9 ± 10.1
20–29 years	504.5 ± 32.1	3016.6 ± 221.3	333.8 ± 24.9	31.6 ± 3.2	54.1 ± 6.5
30–39 years	501.5 ± 30.4	2944.7 ± 205.9	345.1 ± 30.7	33.5 ± 3.4	50.3 ± 6.2
40–49 years	509.4 ± 33.6	2865.2 ± 237.7	351.6 ± 32.1	32.9 ± 3.1	50.3 ± 4.8
50–59 years	505.4 ± 32.2	2773.7 ± 256.5	365.3 ± 37.6	33.9 ± 2.4	50.1 ± 5.0
≥60 years	502.9 ± 29.4	2660.0 ± 355.3	379.8 ± 51.3	35.4 ± 3.4	49.1 ± 6.5
All	504.2 ± 30.7	2907.1 ± 290.9	348.5 ± 38.4	32.9 ± 3.6	51.8 ± 7.2
*p*-Value	0.83	< 0.001	< 0.001	< 0.001	< 0.001
2.5th percentile	449.6	2165.3	293.0	26.0	38.3
97.5th percentile	566.0	3414.4	465.1	40.3	66.4
SW test (p-Value)	0.97 (< 0.001)	0.99 (0.012)	0.92 (< 0.001)	0.99 (0.15)	0.97 (< 0.001)
Skewness	− 0.29	− 0.52	+ 1.28	+ 0.23	+ 0.001

*CCT* Centra corneal thickness; *CECD* Corneal endothelial cell density; *CV* Coefficient of variation in cell size; *HEX* Hexagonality

CCT was comparable in all age bands (*p* = .83), indicating stability with age. On the contrary, CECD and hexagonality decreased whereas cell size and coefficient of variation (CV) increased significantly with age (all *p* < .001). These trends are noted in Table 2. CCT and CEC parameters were comparable in males and females in the study population as a whole and in each of the age bins, except for a marginal but significantly larger cell size in females than males in subjects aged 60 years and beyond (Table 3).

**Table 3:** Central corneal thickness and corneal endothelial cell morphometry in men and women stratified in age groups

Age groups	Men	Women	***p*** value
All combined			
CCT	504.9 ± 33.6	503.6 ± 28.3	0.73
CECD	2917.1 ± 253.5	2899.2 ± 317.8	0.61
Cell size	344.8 ± 30.0	351.3 ± 43.8	0.16
CV	32.6 ± 3.7	33.0 ± 3.5	0.32
HEX	52.1 ± 6.9	51.6 ± 7.4	0.59
10–19 years			
CCT	497.5 ± 39.9	504.4 ± 17.5	0.39
CECD	3073.9 ± 234.7	3122.3 ± 255.9	0.50
Cell size	327.3 ± 26.1	323.3 ± 27.9	0.61
CV	29.7 ± 3.9	31.1 ± 3.5	0.20
HEX	57.8 ± 11.8	54.9 ± 8.9	0.31
20–29 years			
CCT	501.6 ± 35.0	507.4 ± 29.4	0.53
CECD	2975.2 ± 186.5	3056.5 ± 247.4	0.20
Cell size	337.9 ± 21.4	329.9 ± 27.8	0.27
CV	31.1 ± 2.7	32.0 ± 3.6	0.31
HEX	53.3 ± 5.5	54.8 ± 7.3	0.43
30–39 years			
CCT	506.2 ± 32.7	497.4 ± 28.3	0.32
CECD	2979.1 ± 176.9	2915.3 ± 226.9	0.28
Cell size	336.9 ± 21.3	352.0 ± 35.9	0.08
CV	33.2 ± 3.0	33.8 ± 3.7	0.53
HEX	50.9 ± 4.6	49.7 ± 7.3	0.48
40–49 years			
CCT	512.8 ± 32.6	505.9 ± 34.9	0.49
CECD	2888.6 ± 235.2	2846.7 ± 244.1	0.61
Cell size	350.0 ± 32.6	353.2 ± 32.2	0.74
CV	32.9 ± 3.2	32.9 ± 3.1	0.94
HEX	50.9 ± 4.0	49.8 ± 5.6	0.46
50–59 years			
CCT	507.3 ± 30.7	504.5 ± 33.3	0.79
CECD	2798.4 ± 193.8	2761.7 ± 284.3	0.67
Cell size	359.5 ± 25.3	368.0 ± 42.4	0.85
CV	33.9 ± 2.9	33.9 ± 2.2	0.98
HEX	50.3 ± 4.4	49.9 ± 5.4	0.49
≥60 years			
CCT	503.6 ± 32.7	502.4 ± 26.7	0.90
CECD	2740.2 ± 344.6	2583.9 ± 357.0	0.17
Cell size	363.4 ± 37.9	395.5 ± 58.0	0.049
CV	35.4 ± 3.7	35.3 ± 3.2	0.88
HEX	48.9 ± 6.0	49.4 ± 7.1	0.80

*CCT* Central corneal thickness; *CECD* Corneal endothelial cell density; *CV* Coefficient of variation; *HEX* Hexagonality

### Correlation of central corneal thickness and corneal endothelial cell parameters with demographic and clinical parameters

**Fig. 2:**
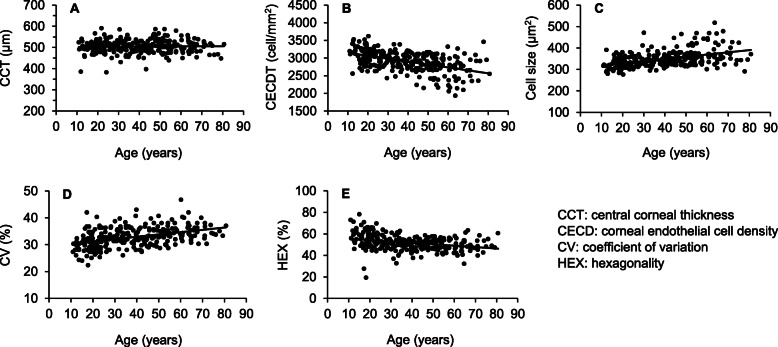
Correlation with regression lines of central cornea thickness (**a**), corneal endothelial cell density (**b**), cell size (**c)**, coefficient of variation in cell size (**d)**, and hexagonality (**e**) with age

Table 4 provides the results of the correlation of CCT and CEC parameters with demographic and clinical variables. CCT only correlated weakly with BMI (*r* = − 0.12, *p* = .046). CECD and hexagonality correlated with age, BMI, and OPP (all *p* < .001). Both cell size and its CV also correlated positively with age and BMI (all *p* ≤ .004). In addition, CECD, cell size, and CV were all weakly but significantly related to IOP (all *p* ≤ .04). The correlations of CCT, CECD, cell size and CV with age are illustrated in Fig. 2. When similar analyses were performed separately in younger (< 50 years old, *n* = 196) and older subjects (≥50 years old, *n* = 82) (data not shown in Table 4), all correlations remained non-significant for CCT in both groups and none was significant in old subjects for CECD (all *p* > .05). In young subjects (Table 4), CECD remained significantly related to age (*p* < .001), BMI (*p* = .02), OPP (*p* = .018), but no longer correlated with IOP (*p* = .46). Hexagonality also still correlated with age (*p* < .001), BMI (*p* = .014) and OPP (*p* = .044. Cell size was no longer related to IOP, but its correlation with age was significant (*p* < .001), BMI (*p* = .04) and OPP (*p =* .02). In this same subgroup CV showed significant correlations only with age (*p* < .001) and BMI (*p* = .01).

**Table 4:** Correlation of CCT and CEC parameters with demographic and clinical variables

Variables	Pearson’s correlation coefficient (***p***-value)					
	Age	IOP	SE	CDR	BMI	OPP
*Full set*						
CCT	0.024 (0.69)	0.03 (0.62)	0.046 (0.44)	0.01 (0.85)	−0.12 (0.046)*	−0.084 (0.16)
CECD	− 0.49 (< 0.001)*	− 0.13 (0.029)*	0.023 (0.71)	− 0.012 (0.85)	− 0.20 (0.001)*	−0.28 (< 0.001)*
Cell size	0.45 (< 0.001)*	0.12 (0.04)*	−0.031 (0.61)	0.041 (0.50)	0.18 (0.004)*	0.29 (< 0.001)*
CV	0.42 (< 0.001)*	0.13 (0.027)*	−0.034 (0.58)	0.007 (0.91)	0.19 (0.002)*	0.10 (0.08)
HEX	−0.32 (< 0.001)*	− 0.091 (0.13)	− 0.047 (0.43)	0.056 (0.35)	− 0.17 (0.004)*	−0.16 (0.009)*
*Subject < 50 years*						
CCT	0.08 (0.25)	0.091 (0.20)	0.097 (0.18)	−0.03 (0.66)	−0.12 (0.09)	− 0.08 (0.28)
CECD	−0.40 (< 0.001)*	− 0.05 (0.46)	0.01 (0.90)	0.06 (0.44)	− 0.17 (0.02)*	− 0.17 (0.018)*
Cell size	0.36 (< 0.001)*	0.01 (0.90)	0.006 (0.94)	−0.03 (0.68)	0.14 (0.04)*	0.17 (0.02)*
CV	0.32 (< 0.001)*	0.10 (0.16)	−0.04 (0.60)	−0.07 (0.32)	0.18 (0.01)*	0.05 (0.48)
HEX	−0.33 (< 0.001)*	− 0.04 (0.60)	− 0.008 (0.92)	0.07 (0.32)	− 0.18 (0.014)*	−0.14 (0.044)*

*CCT* Centra corneal thickness; *CECD* Corneal endothelial cell density; *CV* Coefficient of variation in cell size; *HEX* Hexagonality; *IOP* Intraocular pressure; *SE* Spherical equivalent; *CDR* Cup-to-disc ratio; *BMI* Body mass index; *OPP* Ocular perfusion pressure; *denotes significant correlation

### Factors associated with central corneal thickness and corneal endothelial cell density

Univariable and multivariable linear regression analyses were performed to determine factors that were independently associated with CCT and CECD. Table 5 shows only the factors that showed significant association with these two corneal parameters. BMI was the only factor that showed significant association with CCT in univariable analysis. In this same analysis, CECD was associated with age (β = − 8.3; 95% CI: − 10.0 to − 6.5; *p* < .001), weight (β = − 2.9; 95% CI: − 5.1 to − 0.7; *p* = .011), BMI (β = − 11.3; 95% CI: − 17.8 to − 4.7; *p* = .001), IOP (β = − 13.7; 95% CI: − 25.9 to − 1.4; *p* = .029), SBP (β = − 4.4; 95% CI: − 6.2 to − 2.6; *p* < .001), DBP (β = − 5.1; 95% CI: − 7.7 to − 2.5; *p* < .001), and OPP (β = − 9.2; 95% CI: − 12.9 to − 5.4; *p* < .001). In multivariable analysis, BMI remained the sole variable associated with CCT and age emerged as the sole variable significantly associated with CECD. With each kg/m^2^ increase in BMI, CCT decreased by 0.72 μm whereas each additional year in age decreased CECD by 8.3 cells/mm^2^ or 0.30%.

**Table 5:** Univariable and multivariate regression analyses for central corneal thickness and corneal endothelium cell density

Parameters	Univariate regression		Multivariate regression	
	β (95% CI)	***p*** value	β (95% CI)	***p*** value
Central Corneal Thickness				
BMI	−0.72 (−1.42 – − 0.014)	0.046	−0.72 (−1.42 – − 0.014)	0.045
Corneal Endothelial Cell Density				
Age, years	−8.29 (−10.04 – −6.53)	< 0.001	−8.28 (− 10.06 – −6.50)	< 0.001

*CI* Confidence interval; *BMI* Body mass index

## Discussion

Measuring CCT in a healthy population could provide references to define any abnormal thinning or thickening and may assist the clinician in diagnosing or staging certain diseases and determining the risk for certain pathologies. The study of morphometric features of CEC may also help determine normal references limits; assess, stratify, or predict the risk and point to the need for preventive measures. Both CCT and CEC morphometry profiles may also be used for monitoring the efficacy of a particular treatment. Our study provides such profiles for CCT and CEC determined with specular microscopy in a 10 to 80 years of age healthy Congolese sample.

Reliable interpretation of biologic measures in people under clinical investigation, including thickness of the whole cornea or individual layers and cell density of corneal layers, must be made on the background of pre-determined reference limits. The most common way to establish reference limits of biologic measures from a healthy population has been to consider the central 95% of the reference population [23]. Doing so in the present study determined that the upper and lower reference limits for CCT were 449.6 μm and 566.0 μm, those for CECD were 2165.3 cells/mm^2^ and 3414.4 cells/mm^2^, respectively. The CCT reference limits are lower than those previously reported among Iranians (476 μm and 612 μm) [24] and Lithuanians (489 μm and 609 μm) [9]. It is important to note that for both CCT and CEC, values outside these limits are not necessarily pathologic or necessarily abnormal in any way other than statistically. The Gutenberg Health Study [25] reported 498 μm and 612 μm as the 5th and 95th percentiles, respectively, which are also greater than corresponding values of 455.3 μm and 556.1 μm in the present study. There currently is no consensus on the best percentile cutoffs for references limits. Which of 2.5th–97.5th and 5th–95th percentiles to use as cutoffs for reference limits is therefore debatable, but the earlier are likely more robust since only 5% versus 10% of the population will have values outside the limits. This reasoning also holds for CECD.

The mean central cornea in the present study was thinner whereas CECD was greater than in most other populations (Supplemental Table 1). Since age is known to affect both CCT and CECD, these comparisons can be misleading because of the differences in ages across study populations. We therefore compared our measurements and CEC counts with those obtained in other populations of similar age ranges. This is unlike previous studies where comparison of CCT and/or CECD with other studies were made without considering the difference in age across studies. When we restricted the comparison to previous studies whose whole populations’ age range was comparable to ours, central cornea in this Congolese sample was as thick as measured in Pakistanis [26] and Mongolians [27], but thinner than reported in Thais [11], Cameroonians [15], Iranians [28], Spaniards [29], and Turkish [30]. Among these studies four of them also reported the morphometry of endothelial cells [11, 26, 29, 30]; and measured lower CECD than we found. CECD in our study population was also higher than reported by Gambato et al. [31] in Italians. In two other studies, one reported similar CECD to ours in Chinese [32] whereas the other found a corneal endothelium with lower cell density in Americans and a higher density in Japanese [33]. When the comparison took into consideration only studies that included subjects 20 years or older (*n* = 227), central cornea in the present study (504.7 ± 31.4 μm) was also thinner and/or CECD (2862.8 cells/mm^2^) higher than found in other populations of a comparable age range in South Africa [4], Nigeria [13, 34], Egypt [35], India [36], Iran [37], Lithuania [38], Malaysia [39], Pakistan [40], the Philippines [41], Spain [42], and the USA [43]. In the South African cohort [4], all three races (Blacks, Whites, and Mixed) had thicker central cornea than we found. In 40 years and older subjects (*n* = 128) the cornea (CCT = 506.7 μm) was as thick reported by Gotkas et al. [44], but thinner than measured in all three ethnicities (Chinese, Indian, and Malay) that participated in the Singapore Epidemiology Eye Diseases Study (SEEDS) [5], Afro-Caribbeans in the Barbados Eye Study [21], Ghanaians in the Tema Eye Survey [10, 45], Japanese the Eye Care Health Project [46], Latinos in the Los Angeles Latino Eye Study (LALES) [47], and South Koreans in the Namil Study [48]. This subset of our participants had about the same CECD as Nepali, Bangladeshi and Indians who were undergoing cataract surgery in South Asia [8]. Although CCT (504.2 μm) of those 50 years and older in the present study (*n* = 82) mirrored that of participants in the Nakuru Eye Disease Cohort Study [49], it was thinner than found in the Yunnan Minority Eye Studies [7], the Reykjavik Eye Study [50], and the Rotterdam Study [51]. This pattern is similar to what we observed after comparison with subjects of African and those of European heritage drawn from the African Descent and Glaucoma Evaluation Study (ADAGES) [22], participants of the Gutenberg Health Study [25], as well as other studies [17, 46]. While it has consistently been shown that people of African descent have thinner corneas than those of other ethnicities, it was noteworthy that CCT in the present study was even thinner than measured in other SSA countries [4, 10, 13, 15, 18, 45] and other people of African heritage outside Africa [21, 22]. Also noteworthy was that CECD was higher compared to values found in other populations. While several factors may account to various degrees for these discrepancies, the genetic heterogeneity across populations is likely a major one. Indeed, several heritability studies have provided evidence of strong relationships between genetic components with both CCT and CECD [2, 3]. Though it may be tempting to think that this explanation holds only for the difference between people of African descent and those of other ethnicities (i.e. Europeans and Asians), it is important not to lose sight of the large genetic heterogeneity within SSA. Thus, this may account for the difference in CCT and/or CECD between our population and Cameroonians, Ghanaians, Nigerians, South Africans, and Sudanese. Our observation of thinner central cornea and higher CECD compared to Caucasians and Asians from prior studies concurs with previously reported differences in these measures between different racial and ethnic groups [4, 5, 7, 14, 21, 22, 33]. These discrepancies ultimately support the establishment of reference data in different populations. Because most past studies used ultrasound pachymetry, considered as the gold standard for measuring CCT, we wondered whether the difference in devices could explain the discrepancy between our measurements and those of others. A review a past studies that directly compared measurements acquired by specular microscopy and ultrasound pachymetry in people of European decent revealed inconsistent findings. While most of those studies found significantly thinner corneas with specular microscopy than ultrasound pachymetry, some reported either the opposite or equivalence of measurements. Interestingly, corneas in our subjects were still thinner than those measured with ultrasound pachymetry in older non-Black subjects from past studies. Thus, though the difference in working principles between devices contributes to the discrepancy, biological factors likely account for much of the variability.

The comparison of CCT and CECD between men and women has generated contradictory findings across studies. We observed that the two sexes had comparable CCT and CECD, in agreement with findings of HBS in [13, 15, 18, 35] and outside Africa [9, 30, 44]. Conversely, the Tema Eye Survey in Ghana [10] and four PBS out of Africa [46–48, 52] found thicker corneas in men than women. CECD was comparable in men and women in some HBS [30, 32, 37–39, 44], but significantly differed between them in others, with most studies revealing higher CECD in women than men [8, 12, 41, 53]. Taken together, our findings and those from other studies suggest a possible contribution of other factors such as ocular biometric and anthropometric characteristics, and/or environmental conditions may explain the differences in CCT and CECD between men and women [54].

Contrary to studies that have reported negative correlation between CCT and age, we found stable CCT with aging. While one may argue that the size of our sample and the cross-sectional design of our study may have contributed to this outcome, a similar observation was made by large PBS such as the Gutenberg Health Study [25], the Teheran Eye Study [28], the Reykjavik Eye Study [50], the Rotterdam Study [51], as well as HBS with larger study populations than ours [18, 43]. Siu and Herse [55] investigated the relationship of central, mid-peripheral and peripheral corneal thickness with age in 108 normal subjects aged 15 to 75 years. They found no relationship with age in any of the corneal locations and explained the outcome by the small sample size. They suggested increasing the sample size to 480 after running a power analysis to reach statistical significance level. However, this contention is not supported by the above large PBS. Unlike CCT, CECD decreased significantly with age, a finding that has been consistent in previous studies.

The relationship between CCT and IOP has been extensively investigated in the past. In contrast to the significant positive relationship between CCT and IOP from most studies [15, 25, 47, 48, 51, 52, 56], we surprisingly observed that CCT was unrelated to IOP. The most plausible explanation is that, although IOP rose significantly with age (*r* = 0.33, *P* < 0.001), the increase was not sufficient to affect the CCT. As such, our study mirrors the Barbados Eye Studies where the relationship between CCT and IOP existed only in the 2.2% of European but not the 93.2% of African descent participants [21]. Clinically, this finding suggests that IOP should not be corrected for CCT in this population, as also suggested by others [21, 57]. The lack of CCT-IOP relationship contrasted with significant decrease in CECD with rising IOP in the present study. Thus, IOP affects CCT and CECD differently, with CECD being more sensitive to IOP elevation than CCT. Of note, absence of CECD-IOP relationship has also been reported [20, 53]. While the reason for this discrepancy among studies may be multifactorial, the cohort effect cannot be neglected even though so far studies have not been considering this issue.

Information on the relationship of CCT and CECD with BMI is scare in the literature. We report herein that CCT and BMI were negatively related, differing with lack of relationship reported in two previous studies [5, 58], and a positive correlation observed in the Singapore Malay Eye Study [54], and the Central India Eye and Medical Study [52]. Regarding CECD, a significant positive correlation with BMI have been mentioned [58]. Since IOP rose with increasing BMI in our population (*r* = 0.18, *P* = 0.002) as consistently shown in epidemiologic studies, it is possible that the relationship between BMI and CECD is in fact driven by IOP. A similar hypothesis, previously put forth by others for the association of higher BMI with thicker corneas, could not be verified in this study.

Estimating the rate of CCT thinning and CECD loss in healthy subjects is important to determine the upper limits beyond which rates may be considered abnormal. Such rates may also help identify subjects at risk of developing certain diseases. In other instances, they may be markers for disease progression and prompt the clinician to escalate or completely modify the current treatment. Although CCT was not associated with age in the present study, available data show large variations in CCT rate of thinning with age. For example, rates of 0.42 μm/year and 1.1 μm/year were reported, respectively, in a cross-sectional hospital-based study in Cameroon (*n* = 485, age = 5–79 years) [15] and an 8-year longitudinal population-based study in Ghana (*n* = 758, age ≥ 40 years) [59]. Outside Africa, cross-sectional studies estimated that CCT decayed by 0.28 μm/year among 12–60 years old Turkish [60], 0.48 μm/year in 40 years and older Mongolians [27], 0.58 μm/year of age in 30 years and older Lithuanians [9], 0.5 μm/year in 6 years and older Iranians [61], 0.3 μm/year in a multiethnic population including Caucasians, Chinese, Japanese, Hispanics, Filipinos, and African Americans aged in average 67.3 years [62], and 0.3 μm/year and 0.5 μm/year in 50 years and older Chinese [63]. Regression analysis suggested that CECD decayed at a rate of 8.3 cells/mm^2^ or 0.30% per year of age in the present study, which is comparable to 0.27–0.30% reported in Pakistani [26], Chinese [32], and Indians [36]; higher than 0.23 and 0.25% per year found in Thais [11] and Japanese [64], respectively, but lower than 0.5–0.6% estimated in Chinese [65, 66], New Zealanders [67] and Americans [68].

Our study provides reference data from a homogeneous black population. While it may have some shortcomings generally associated with a HBS, it is important to consider that PBS are not easy to perform in resource-limited settings. In such circumstances, HBS can provide valid data when well designed and executed. In fact, built-in normative databases on most ocular imaging devices are collected in ophthalmology clinics. Strengths of the study include a relatively large sample size with a wide age range. Also particular to this study is that we avoided bias when comparing our findings with those of prior studies by matching age ranges. By only including subjects who were healthy and excluding those with ocular and/or systemic confounding factors, the present study provides what we believe are accurate and reliable CCT and CEC characteristics in healthy Congolese subjects.

In conclusion, this study provides the first characterization of specular microscopy-based CCT and CEC in 10 to 80 years old Congolese healthy subjects. The 2.5th and 97.5th reference limits of CCT were lower whereas such cutoffs for CECD were higher than those from other populations. Mean central cornea was thinner contrasting with higher CECD than previously reported in other populations. CCT was not associated with either age or IOP but was inversely associated with BMI; this observation will need corroboration by other studies in the same population. CECD correlated negatively with age, IOP, BMI and OPP, but was only associated with age. Altogether, this information provides a reference foundation suitable for future comparative studies of CCT and CEC in Congolese subjects.

## Supplementary Information

**Additional file 1: Supplemental Table 1.** Comparison of central corneal thickness and corneal endothelial cell morphometric characteristics across selected studies

## Data Availability

The datasets used and/or analyzed during the current study are available from the corresponding author upon reasonable request.
