# Supplementary material for: Profile of central corneal thickness and corneal endothelial cell morpho-density of in healthy Congolese eyes
Source: BMC Ophthalmol. 2021 Apr 22;21:185. doi: 10.1186/s12886-021-01947-x (PMC8063471; doi:10.1186/s12886-021-01947-x)
Supplement: Supplementary file 1 — Additional file 1: Supplemental Table 1. Comparison of central corneal thickness and corneal endothelial cell morphometric characteristics across selected studies [file 12886_2021_1947_MOESM1_ESM.docx]

**Profile of Central Corneal Thickness and Corneal Endothelial Cell Morpho-Density of in Healthy Congolese Eyes**

Joseph-Theodore K. Kelekele^1^, David L. Kayembe^1^, Jean-Claude Mwanza^1,2^

^1^Department of Ophthalmology, University Hospital of Kinshasa, Kinshasa, Democratic Republic of Congo

^2^Department of Ophthalmology, University of North Carolina at Chapel Hill, Chapel Hill, North Carolina, USA

Corresponding author: Joseph-Theodore Kelekele, Department of Ophthalmology, University Hospital of Kinshasa, Kinshasa, Democratic Republic of Congo.

Email: [jostkelekele@gmail.com](mailto:jostkelekele@gmail.com), tel: +243 998-162-338.

| **Supplemental Table 1** Comparison of central corneal thickness and corneal endothelial cell morphometric characteristics across selected studies | | | | | | | | | | | | | | | | |
| --- | --- | --- | --- | --- | --- | --- | --- | --- | --- | --- | --- | --- | --- | --- | --- | --- |
| **Studies on Corneal Endothelial Cell** | | | | | | | | | |  | **Studies on Central Corneal Thickness** | | | | | |
| **Study** | **Ethnicity** | **N subjects** | **Age**  **(range)** | **Method** | **CCT** | **CECD** | **Cell**  **size** | **CV** | **HEX** |  | **Study** | **Ethnicity** | **N**  **subjects** | **Age**  **(range)** | **Method** | **CCT** |
| Present | Congolese | 278 | 38.9 (10-80) | SM | 504.2 | 2907.1 | 348.5 | 32.9 | 51.8 |  | Present | Congolese | 278 | 38.9 (10-80) | SM | 504.2 |
| Rao et al^36^ | Indian | 537 | 48 (20-78) | SM | 523.3 | 2525 | 403.6 | 35.8 | 57.3 |  | Eballe et al^15^ | Cameroonian | 435 | 31.4 (5-75) | USP | 528.7 |
| Jorge et al^29^ | Spanish | 256 | 43.5 (6-82) | SM | 567.5 | 2526.4 | 404.3 | 33 | 53.9 |  | Iyamu et al^13^ | Nigeria | 130 | 47.8 (20-79) | USP | 548.9 |
| Tunanuvat et al^11^ | Thai | 501 | 43.1 (11-88) | SM | 533.8 | 2732.5 | 369.0 | 37.6 | 49.0 |  | Kim et al^45^ | Ghanaian | 155 | 57 (40-98) | USP | 525.3 |
| Sopapornamorn et al^52^ | Thai | 202 | 45.7 (20-80) | SM | - | 2623.5 | - | 39.4 | 51.5 |  | Sng et al^10^ | Ghanaian | 4737 | 51.9 (≥40) | USP | 533.9 |
| Salih et al^39^ | Malay | 125 | 45.8 (20-87) | SM | - | 2648 | 382.8 | 58.1 | 44.3 |  | Gelaw et al^17^ | Ethiopian | 300 | 42.6 | USP | 518.7 |
| Abdellah et al^35^ | Egyptian | 568 | 49 (20-85) | SM | 514.5 | 2647.5 | 390.6 | 32.3 | 53.8 |  | Baboolal et al^4^ | South African | 402 | 43 (18-94) | PENT | 528.1 |
| Duman et al^30^ | Turkish | 282 | 42 (6-80) | SM | 513 | 2752 | 368 | 44 | 46 |  | Bastawrous et al^49^ | Kenyan | 4114 | (≥50) | ASOCT | 508.1 |
| Goktas et al^44^ | Turkish | 517 | 68.4 (44-90) | SM | 500 | 2258 | 468 | 39.3 | 49.3 |  | Nemesure et al^21^ | Afro-Caribbean | (2120) | ≥40 | USP | 529.3 |
| Islam et al^26^ | Pakistani | 232 | 39.5 (10-80) | SM | 505.7 | 2722.7 | 374.1 | 33.7 | 55.8 |  | Sample et al^22^ | African descent | 393 | 46.2 (≥30) | USP | 533.8 |
| Ewete et al^12^ | Nigerian | 210 | 50.3 (20-93) | SM | - | 2610.3 | 392.2 | 43.9 | 46.5 |  | Sample et al^22^ | European descent | 367 | 49.5 (≥30) | USP | 551.9 |
| Ani et al l^34^ | Nigerian | 480 | 43 (18-91) | SM | - | 2783 | - | - | - |  | Hahn et al^47^ | Latinos | 1578 | 53.9 (≥40) | USP | 546.5 |
| Galgauskas et al^38^ | Lithuanian | 211 | 54.5 (20-89) | SM | 554.6 | 2588.9 | 396.1 | 32.1 | 59.3 |  | Hoffmann et al^25^ | German | 4698 | 56.0 (35-74) | SCH | 554.2 |
| Padilla et al^41^ | Filipino | 320 | 53 (20-86) | SM | - | 2798 | 363.0 | 32.5 | 59.4 |  | Eysteinsson et al^50^ | Islandic | 925 | ≥50 | SCH | 527 |
| Gambato et al^31^ | Italian | 108 | 41.5 (11-74) | SM |  | 2817.8 | - | 35.8 | 52.4 |  | Wolfs et al^51^ | Dutch | 352 | 72 (55-90) | USP | 547.4 |
| Matsuda et al^33^ | Japanese | 60 | (13-83) | SM | - | 3612.4 | 279.6 | 29.5 | - |  | Suzuki et al^46^ | Japanese | 7313 | 56.7 | SM | 517.5 |
| Matsuda et al^33^ | American | 73 | (14-85) | SM | - | 2771.3 | 368.8 | 38.4 | - |  | Pan et al^7^ | Chinese Bai | 2119 | 64.4 (≥50) | USP | 536.4 |
| Snellingen et al^8^ | Nepali | 302 | (40-75) | SM | - | 2634 | - | 39.3 | 34.4 |  | Pan et al^7^ | Chinese Yi | 2202 | 65.0 (≥50) | USP | 532.1 |
| Snellingen et al^8^ | Bengladeshi | 646 | (40-75) | SM | - | 2782 | - | 33.2 | 37.8 |  | Pan et al^7^ | Chinese Han | 2183 | 65.4 (≥50) | USP | 529.6 |
| Snellingen et al^8^ | Indian | 469 | (40-75) | SM | - | 2714 | - | 41.3 | 45.2 |  | Foster et al l^27^ | Mongolian | 1129 | (10-87) | OP | 503.8 |
| Hashemian et al^37^ | Iranian | 525 | 52.2 (20-85) | SM | - | 1961 | 537.0 | 24.1 | - |  | Hashemi et al^28^ | Iranian | 399 | 40.9 (14-81) | USP* | 555.6 |
| Ashraf et al^40^ | Pakistani | 225 | 45.4 (20-70) | SM | - | 2654 | - | - | - |  | Chua et al^5^ | Chinese | 3425 | 59.6 (40-80) | USP | 552.3 |
| Roszkowska et al^42^ | Italian | 204 | (20-83) | SM | - | 2620 | - | - | - |  | Chua et al^5^ | Indian | 4382 | 57.5 (40-80) | USP | 540.4 |
| Yunliang et al^32^ | Chinese | 700 | 44 (10-98) | SM | - | 2932 | 347 | 33 | 59 |  | Chua et al^5^ | Malay | 4410 | 59.1 (40-80) | USP | 540.9 |
| *CCT; central corneal thickness; CECD, corneal endothelial cell density; CV, coefficient of variation; HEX, hexagonality; SM, specular microscopy; USP, ultrasound pachymetry; PENT, pentacam; ASOCT, anterior segment optical coherence tomography; SCH, Scheimpflug imaging; OP, optical pachymetry; *values obtained after converting measurements acquired with Orbscan.* | | | | | | | | | | | | | | | | |
